# The Modifiable Risk Factors of Uncontrolled Hypertension in Stroke: A Systematic Review and Meta-Analysis

**DOI:** 10.1155/2021/6683256

**Published:** 2021-02-24

**Authors:** Arif Setyo Upoyo, Ismail Setyopranoto, Heny Suseani Pangastuti

**Affiliations:** ^1^Faculty of Health Sciences, Jenderal Soedirman University, Indonesia; ^2^Doctoral Program of Nursing, Faculty of Medicine, Public Health, and Nursing, Gadjah Mada University, Indonesia; ^3^Department of Neurology, Faculty of Medicine, Public Health, and Nursing, Gadjah Mada University, Indonesia; ^4^Department of Medical and Surgical Nursing, Faculty of Medicine, Public Health, and Nursing, Gadjah Mada University, Indonesia

## Abstract

**Objective:**

This review aimed at figuring out the risk factors of uncontrolled hypertension in stroke.

**Method:**

This study systematically analyzed the hypertension risk factors available in the ProQuest, EBSCO, and PubMed databases published between 2010 and December 2019. The risk factors' pooled odds ratio (POR) included in this research was calculated using both fixed and random-effect models. The meta-data analysis was processed using the Review Manager 5.3 (Rev Man 5.3).

**Result:**

Of 1868 articles, seven studies were included in this review searched using specific keywords. Based on the analysis results, there were 7 risk factors of uncontrolled hypertension in stroke: medication nonadherence (POR = 2.23 [95% CI 1.71-2.89], *p* = 0.342; *I*^2^ = 6.7%), use of antihypertensive drugs (POR = 1.13 [95% CI 1.19-1.59, *p* = 0.001; *I*^2^ = 90.9%), stage of hypertension (POR = 1.14 [95% CI 1.02-1.27], *p* = <0.001; *I*^2^ = 97.1%), diabetes mellitus (POR = 0.71 [95% CI 0.52-0.99], *p* = <0.001; *I*^2^ = 96.5%), atrial fibrillation (POR = 1.74 [95% CI 1.48-2.04)], *p* = <0.001; *I*^2^ = 93.1%), triglycerides (POR = 1.47 [95% CI 1.23-1.75], *p* = 0.879; *I*^2^ = 0%), and age (POR = 1.03 [95% CI 0.89-1.18], *p* = <0.001; *I*^2^ = 97.5%]. There were no bias publications among studies. Medication nonadherence and triglycerides had homogeneous variations, while the others had heterogeneous variations.

**Conclusion:**

Medication nonadherence, triglycerides, stage of hypertension, atrial fibrillation, and use of antihypertensive drugs significantly affect the uncontrolled hypertension in stroke.

## 1. Introduction

Stroke is one main cause of death and disability in many countries [1]. It was globally reported that, in 2013, there were nearly 25.7 million stroke sufferers and 10.3 million new stroke cases [2]. The prevalence of stroke in Indonesia annually increases. Based on the Basic Health Research conducted in 2013, the prevalence of stroke was 7 per one thousand people of the population. Meanwhile, in 2018, the prevalence of stroke was 10.9 per one thousand people of the population [3].

The risk factors in stroke include both nonmodifiable and modifiable factors. The nonmodifiable factors include age, sex, race/ethnicity, and genetics. Meanwhile, the modifiable factors include hypertension, diabetes, atrial fibrillation, dyslipidemia, diet, physical activity, obesity, metabolic syndrome, alcohol consumption, and smoking [4, 5]. Patients with hypertension have 2.87 times of risks to experience stroke [6]. The other studies also stated that the prevalence of stroke in patients with hypertension aged 50 years was 20% of the population with a risk ratio of 4 and the prevalence continuously increases along with the increasing number of age [7].

Controlling the modifiable risk factors in stroke is greatly important to prevent from stroke. The problems faced in controlling the risk factors in stroke include inaccurate knowledge [8], nonoptimal adherence [9, 10], and less awareness to the stroke risks [11].

This study determined the uncontrolled hypertension risk factors in stroke using some studies through a systematic review and meta-analysis to result in stronger conclusions.

## 2. Method

### 2.1. Research Design and Samples

This research was quantitatively conducted with a meta-analysis research design. The review followed the Preferred Reporting Items for Systematic Reviews and Meta-Analyses (PRISMA) guidelines [12]. Meta-analysis was used to figure out the risk factors for the uncontrolled hypertension in stroke. The research samples were the research articles on hypertension risk factors available in the ProQuest, EBSCO, and PubMed databases published between 2010 and December 2019. The inclusion criteria were studies on the uncontrolled hypertension risk factors in stroke with a control case or cohort study. Meanwhile, the exclusion criteria were those not available in the full-text forms.

### 2.2. Operational Definitions

The variables in this study including the independent variables were the modifiable factors consisting of physical activity, obesity, smoking, alcohol consumption, sodium consumption, saturated fat consumption, diabetes, hypercholesterolemia, triglycerides, knowledge, therapy adherence, and atrial fibrillation. Meanwhile, the nonmodifiable risk factors included age, sex, and family history of hypertension. The dependent variable in this research was the uncontrolled hypertension in stroke. It was considered uncontrolled if the SBP is ≥140 mm Hg and/or the DBP is ≥90 mm Hg [13].

### 2.3. Research Procedures

**Figure 1:**
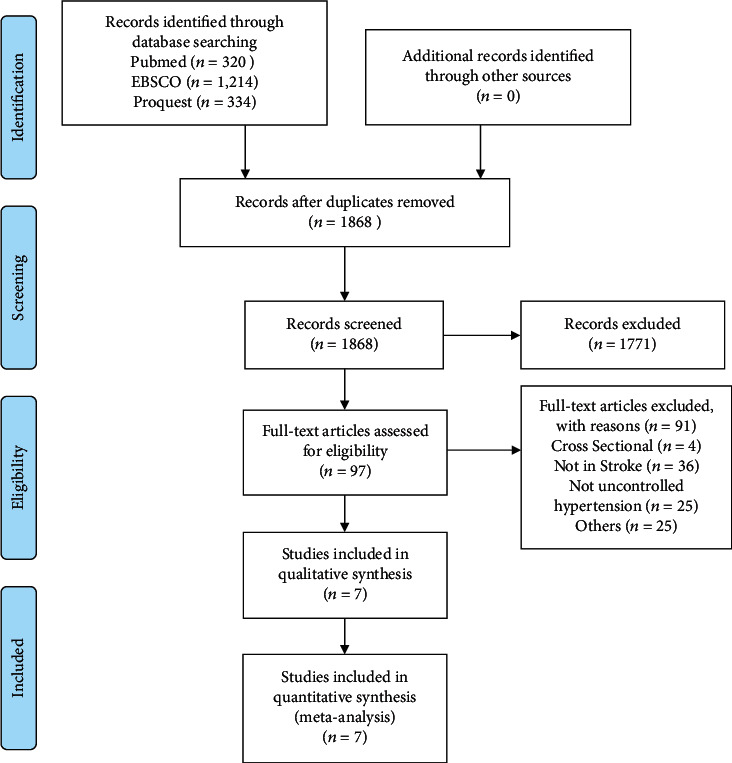
Flow diagram on the selection processes examining the risk factors of uncontrolled hypertension in stroke.

This study was conducted by identifying the research articles on the uncontrolled hypertension risk factors in stroke available in the PubMed, ProQuest, and EBSCO databases published between 2010 and December 2019 (Figure 1).

Searching was made by entering the following keywords: ((uncontrolled blood pressure OR uncontrolled hypertension) AND (risk factors OR age OR sex OR family history of hypertension OR physical activity OR activity OR body mass index OR obesity OR smoking OR tobacco use OR cigarette OR alcohol consumption OR unhealthy diet OR diet OR trans-fat OR saturated fat OR sodium consumption OR salt consumption OR diabetes OR hypercholesterolemia OR knowledge OR stress OR anxiety OR adherence OR self-care OR self-management OR compliance of therapy) AND (Stroke)).

Searching was limited only for the English language articles. The article type was limited only to the original journal articles. The publication period was limited only from 2010 to December 2019. Articles with potentially relevant titles were then reviewed based on their abstracts, while the irrelevant ones were excluded. The articles with potentially relevant abstracts were then fully reviewed. Meanwhile, the irrelevant ones were excluded. Furthermore, an article will be excluded if the research design is not a case control or cohort study and the variable is not the uncontrolled hypertension in stroke.

### 2.4. Data Analysis

The data were analyzed to obtain the value of pooled odds ratio (POR), that is, the combined odds ratio values from the related studies. The data were analyzed using the Mantel-Haenszel method with both fixed and random-effect model proposed by DerSimonian-Laird. The data were then analyzed using the Review Manager 5.3 (Rev Man 5.3).

## 3. Results

The searching made using specific keywords was to identify 1868 articles and then review the article titles, abstracts, and full-texts. Irrelevant articles were then excluded. The included seven studies were reviewed since correlated with the risk factors of the uncontrolled hypertension in stroke (Table 1). The research variables were systematically reviewed, while the meta-analysis was the modifiable risk factors including BP medication nonadherence, stage of hypertension, and the use of antihypertensive drugs.

**Figure 2:**
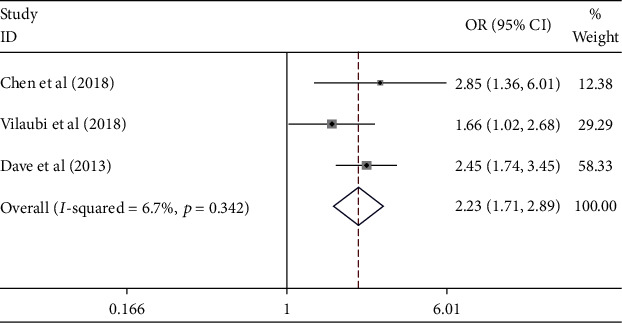
Effect size of medication nonadherence on the uncontrolled hypertension.

**Figure 3:**
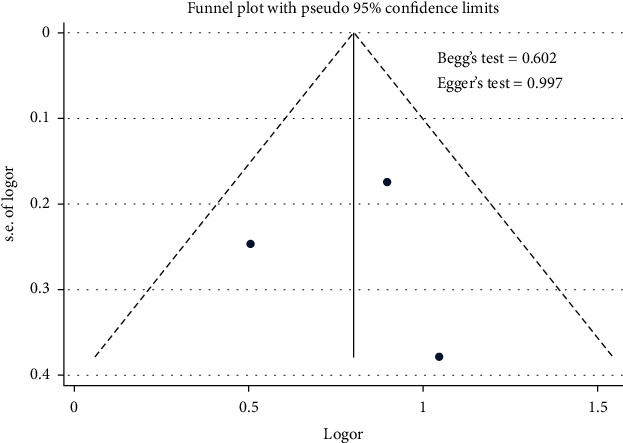
Funnel plot role of medication nonadherence on the uncontrolled hypertension.

The effect of medication nonadherence on the uncontrolled hypertension was presented in Figure 2. Figure 2 shows that the medication nonadherence contributed to the uncontrolled hypertension [pooled odds ratio (POR) = 2.23 (95%CI 1.71 − 2.89)]. There was heterogeneity among studies regarding to the role of medication nonadherence factor on the uncontrolled hypertension (*p* heterogeneity = 0.342; *I*^2^ = 6.7%). It indicates that the variation among studies was homogeneous. The funnel plots to identify the publication bias among studies related to the medication nonadherence factor on the uncontrolled hypertension were presented in Figure 3. Figure 3 shows that there was no significant publication bias for the studies related to the medication nonadherence factor on the uncontrolled hypertension, respectively, based on the Egger's test (*p* = 0.997) and Begg's test (*p* = 0.602).

**Figure 4:**
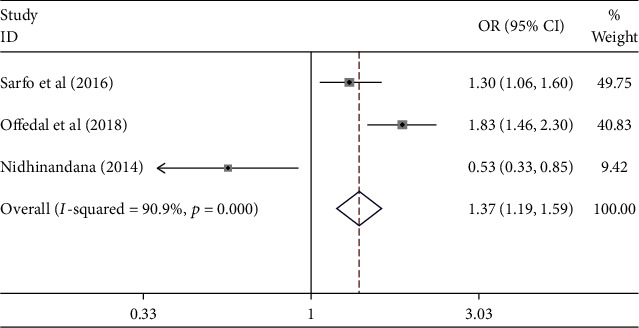
Effect size of use of antihypertensive drugs on the uncontrolled hypertension.

**Figure 5:**
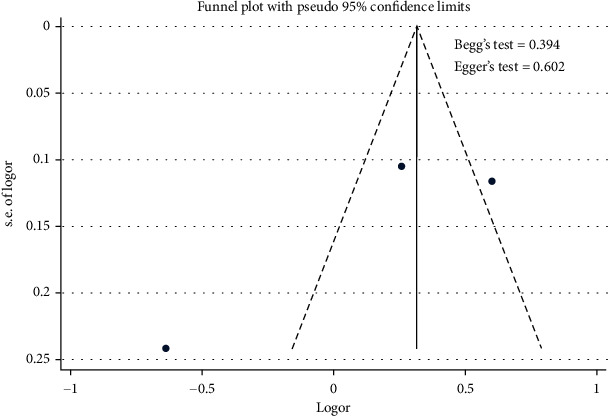
Funnel plots role of use of antihypertensive drugs on the uncontrolled hypertension.

Figure 4 shows that the use of hypertensive drugs contributed to the uncontrolled hypertension [pooled odds ratio (POR) = 1.37 (95%CI 1.19 − 1.59)]. There was heterogeneity among studies regarding the role of the use of antihypertensive drugs on the uncontrolled hypertension (*p* heterogeneity = 0.001; *I*^2^ = 90.9%). It shows that the variation among studies was heterogeneous. The funnel plots to identify the publication bias among studies related to the use of antihypertensive drugs on the uncontrolled hypertension were presented in Figure 5. Figure 5 shows that there was no significant publication bias among studies related to the use of antihypertensive drugs on the uncontrolled hypertension, respectively, based on the Egger's test (*p* = 0.602) and Begg's test (*p* = 0.394).

**Figure 6:**
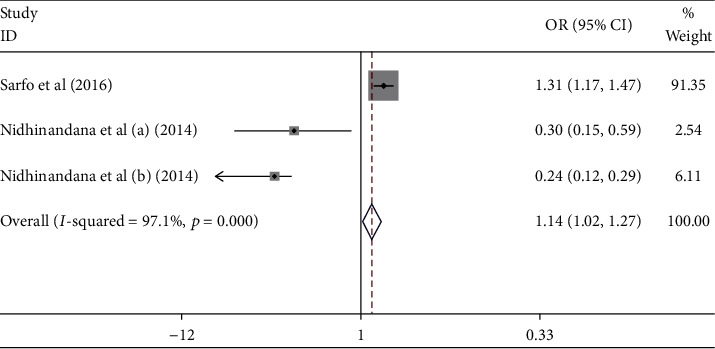
Effect size of hypertension stage on the uncontrolled hypertension.

**Figure 7:**
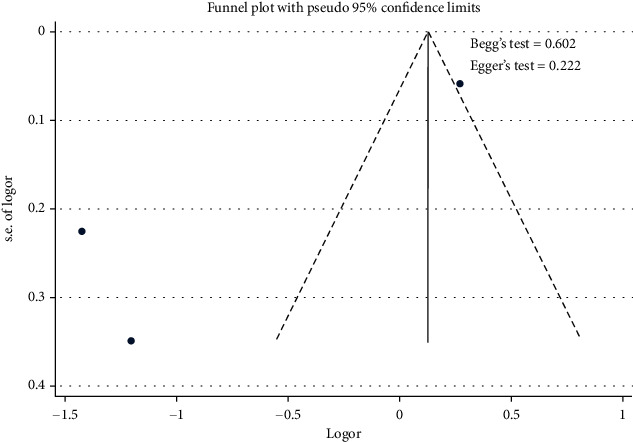
Funnel plots role of hypertension stage on uncontrolled hypertension.

Figure 6 shows that the stage of hypertension contributed to the uncontrolled hypertension [pooled odds ratio (POR) = 1.14 (95%CI 1.02 − 1.27)]. There was heterogeneity among studies regarding the role of stage of hypertension on the uncontrolled hypertension (*p* heterogeneity = 0.000; *I*^2^ = 97.1%). This indicates that the variation between studies was heterogeneous. The funnel plots to identify publication bias between studies related to the stage hypertension and uncontrolled hypertension were presented in Figure 7. Figure 7 shows that there was no significant publication bias on the studies related to the stage of hypertension on the uncontrolled hypertension, respectively, based on the Egger's test (*p* = 0.222) and Begg's test (*p* = 0.602).

**Figure 8:**
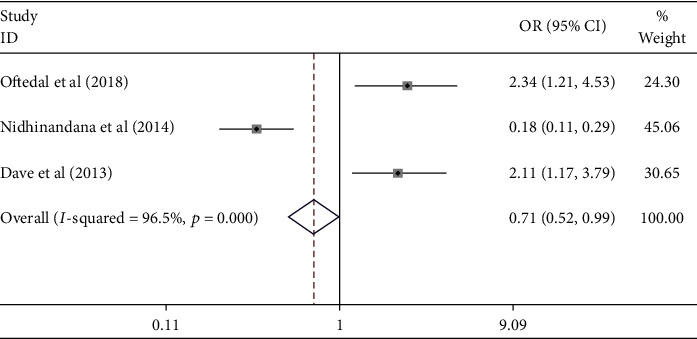
Effect size of diabetes on the uncontrolled hypertension.

**Figure 9:**
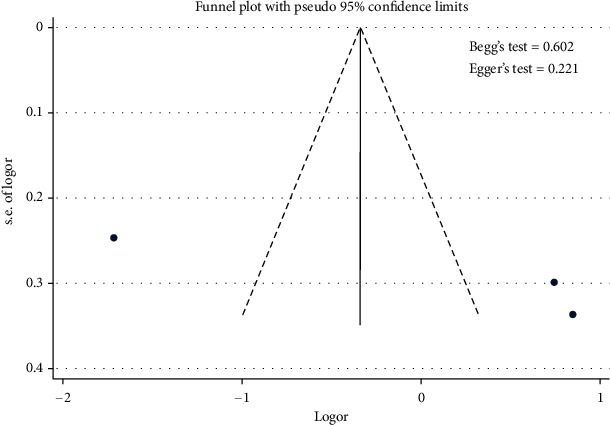
Funnel plots role of diabetes on the uncontrolled hypertension.

Meta-analysis results showed the effect of diabetes on the uncontrolled hypertension (Figure 8). Figure 8 shows that stage of diabetes did not contribute to the uncontrolled hypertension [pooled odds ratio (POR) = 0.71 (95%CI 0.52 − 0.99)]. There was heterogeneity among studies regarding the role of diabetes on the uncontrolled hypertension (*p* heterogeneity = <0.001; *I*^2^ = 96.5%). This indicates that the variation between studies was heterogeneous. The funnel plots to identify the publication bias among studies related to diabetes on the uncontrolled hypertension were presented Figure 9. Figure 9 shows that there was no significant publication bias among studies related to diabetes on the uncontrolled hypertension, respectively, based on the Egger's test (*p* = 0.221) and Begg's test (*p* = 0.602).

**Figure 10:**
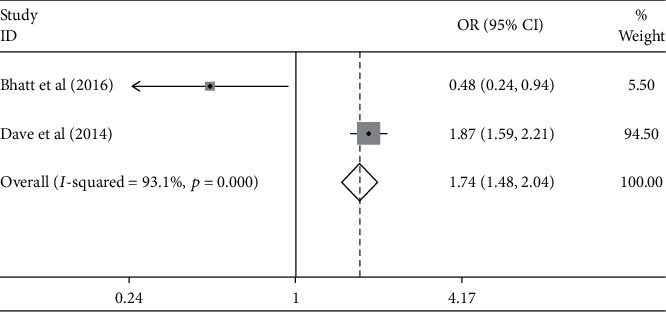
Effect size of atrial fibrillation on the uncontrolled hypertension.

**Figure 11:**
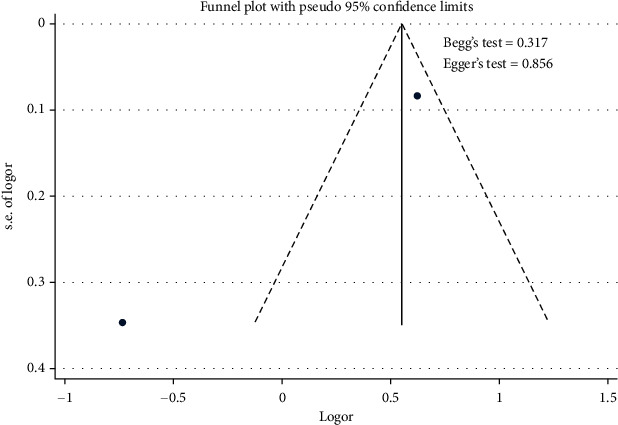
Funnel plots role of atrial fibrillation on the uncontrolled hypertension.

The meta-analysis results showed the effect of atrial fibrillation on the uncontrolled hypertension (Figure 10). Figure 10 indicates that the atrial fibrillation contributed to the uncontrolled hypertension [pooled odds ratio (POR) = 1.74 (95%CI 1.48 − 2.04)]. There was heterogeneity among studies regarding the role of atrial fibrillation factor on the uncontrolled hypertension (*p* heterogeneity = 0.000; *I*^2^ = 93.1%) This shows that the variation among studies was heterogeneous. The funnel plots to identify publication bias among studies related to the atrial fibrillation on the uncontrolled hypertension were presented in Figure 11. Figure 11 shows that there was no significant publication bias among the studies related to the atrial fibrillation on the uncontrolled hypertension, respectively, based on the Egger's test (*p* = 0.856) and Begg's test (*p* = 0.317).

**Figure 12:**
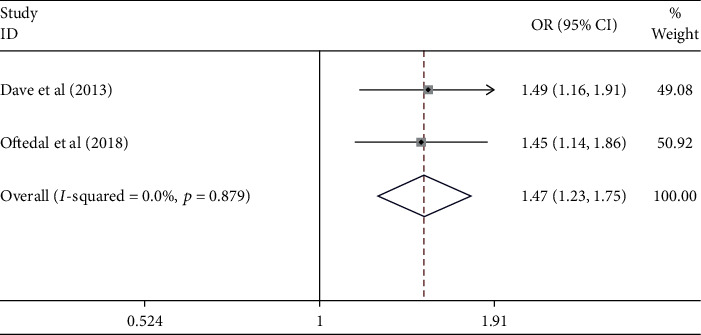
Effect size of triglycerides on the uncontrolled hypertension.

**Figure 13:**
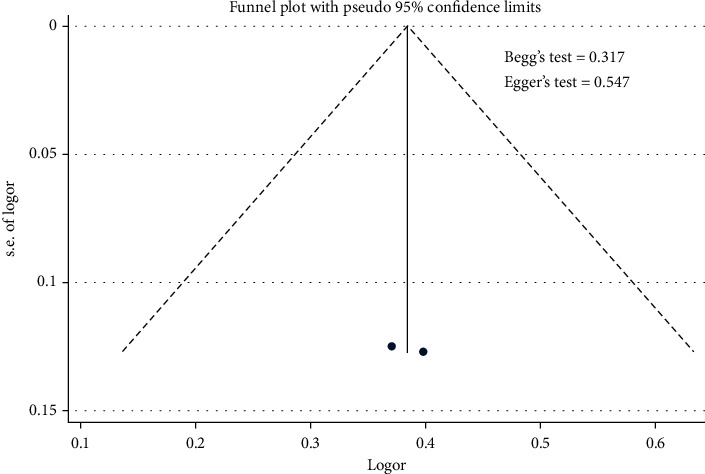
Funnel plots role of triglycerides on the uncontrolled hypertension.

Figure 12 shows that triglyceride contributed to the uncontrolled hypertension [pooled odds ratio (POR) = 1.47 (95%CI 1.23 − 1.75)]. There was heterogeneity among studies regarding the role of triglyceride factor on the uncontrolled hypertension (*p* heterogeneity = 0.879; *I*^2^ = 0%). This indicates that the variation among studies was homogeneous. The funnel plots to identify the publication bias among studies related to the triglyceride factor on the uncontrolled hypertension were presented in Figure 13. Figure 13 shows that there was no significant publication bias among the studies related to the triglyceride factor on the uncontrolled hypertension, respectively, based on the Egger's test (*p* = 0.547) and Begg's test (*p* = 0.317).

**Figure 14:**
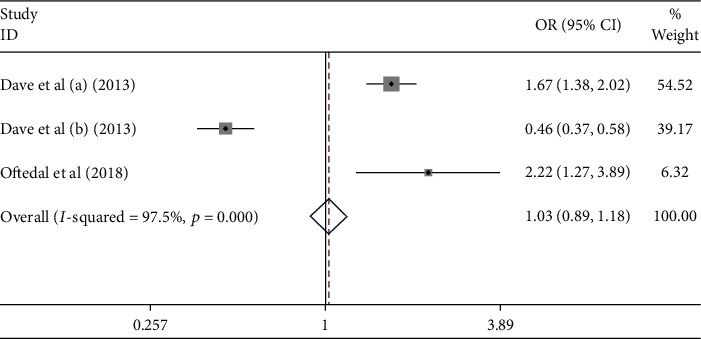
Effect size of age on the uncontrolled hypertension.

**Figure 15:**
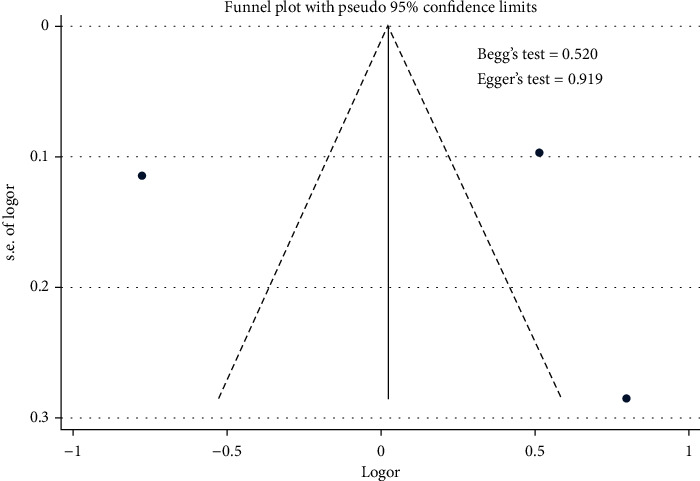
Funnel plots role of age on the uncontrolled hypertension.

The meta-analysis results showed that age was the nonmodifiable risk factor of the uncontrolled hypertension (Figure 14). Figure 14 indicates that age did not contribute to the uncontrolled hypertension [pooled odds ratio (POR) = 1.03 (95%CI 0.89 − 1.18)]. There was heterogeneity among studies regarding the role of age on the uncontrolled hypertension *p*( heterogeneity = <0.001; *I*^2^ = 97.5%). This indicates that the variation among studies was heterogeneous. The funnel plots were to identify the publication bias among studies related to the age on the uncontrolled hypertension (Figure 15). Figure 15 shows that there was no significant publication bias among studies related to the age on the uncontrolled hypertension, respectively, based on the Egger's test (*p* = 0.919) and Begg's test (*p* = 0.520).

## 4. Discussion

The meta-analysis results showed that the modifiable risk factors of the uncontrolled hypertension in stroke was treatment nonadherence with the highest POR value (POR = 2.23 [95%CI 1.71 − 2.89], *p* = 0.342; *I*^2^ = 6.7%) followed by atrial fibrillation (POR = 1.74 [95%CI 1.48 − 2.04]), triglycerides (POR = 1.47 [95%CI 1.23 − 1.75], *p* = 0.879; *I*^2^ = 0%, *p* = <0.001; *I*^2^ = 93.1%), stage of hypertension (POR = 1.14 [95%CI 1.02 − 1.27], *p* = <0.001; *I*^2^ = 97.1%), and use of antihypertensive drugs (POR = 1.13[95%CI 1.19 − 1.59, *p* = 0.001; *I*^2^ = 90.9%). Medication nonadherence and triglycerides had the homogeneous variations among the studies, while the others had heterogeneous ones.

Based on the research results, 80.73% of stroke patients had the uncontrolled blood pressure, and 75.11% had the antihypertensive medication nonadherent. The main reasons to the treatment nonadherence were forgetfulness (58.08%), lack of confidence in obtaining the long-term antihypertensive treatment (27.75%), and not realizing the importance of long-term treatment (24.75%) [14]. A high prevalence of uncontrolled hypertension and medication nonadherence was also shown in the other studies with 67.1% of patients had the uncontrolled blood pressure and 56.1% had the therapy nonadherence [15]. However, other studies showed that the prevalence of uncontrolled hypertension in stroke patients was lower with 43.5% had the uncontrolled systolic blood pressure (SBP), 22.8% had the uncontrolled diastolic blood pressure (DBP), and 18.5% had the combination of SBP and DBP. One risk factor in this study was related to the medication nonadherence which became the obstacles to adherence including forgetfulness, believing in the unwanted side effects, health irresponsibility, absence of symptoms, and problems with access to treatments and drugs [16].

The second factor influencing the uncontrolled hypertension in this study was atrial fibrillation. The prevalence of atrial fibrillation in stroke patients in some studies was 4.3% [16]. Meanwhile, the other studies included in this study had the prevalence of atrial fibrillation in stroke patients by 8.6% [17]. Atrial fibrillation is one of the most common cardiac arrhythmias found to increase the risks of heart failure and stroke as well as to increase the mortality in cardiovascular diseases [18, 19]. In some studies on patients with hypertension, the prevalence of atrial fibrillation was higher in the uncontrolled hypertension patients than that in the controlled hypertension [20]. The risk of atrial fibrillation was decreased with the use of antihypertensive drugs, such as beta blockers and angiotensin converting enzyme inhibitors (ACEI) [21–23].

Triglycerides affect the uncontrolled hypertension. High triglyceride levels can lead to the thickening walls of blood vessels which can increase the risk of stroke and heart disease since related to the occurring arterial plaque as a covariate of the uncontrolled hypertension in stroke patients [24].

Stage of hypertension was the other factor having a significant effect in this study. Every 10 mmHg increase in systolic blood pressure (SBP) will also increase the risk of uncontrolled hypertension in the poststroke patients [25]. Other studies explained that stage of hypertension was associated with the controlled blood pressure (stage I at the discharge of OR = 0.30 [95%CI = 0.15 − 0.59] and stage II at the discharge of OR = 0.24 [95%CI = 0.12 − 0.49]) [26].

The use of antihypertensive drugs also influenced the uncontrolled hypertension. The optimal blood pressure target for the secondary prevention in ischemic stroke patients was still debatable [27]. The results of study conducted by Oftedal et al. [24] revealed that the patients with uncontrolled hypertension used a higher number of antihypertensive drugs than those with the controlled hypertension, although the average number of drugs was <2 in both groups, less than 10% used 3 or more antihypertensive drugs. Thus, the systematic antihypertensive treatments given to the ischemic stroke patients with hypertension were more potential to reduce the high risk of the occurring vascular disorders [28].

## 5. Conclusion

Medication nonadherence, stage of hypertension, atrial fibrillation, triglycerides, and use of antihypertensive drugs had a significant effect on the uncontrolled hypertension in stroke. The medication nonadherence and triglycerides strongly affected the uncontrolled hypertension in stroke. To improve the controlled blood pressure in stroke patients, some efforts are greatly required to overcome the obstacles to adherence.

## Figures and Tables

**Table 1:** Study characteristics.

First author (year)	Type of study	Period of research	Total subject	Total cases	Risk of factor	OR/RR	CI(95%)	Impact
Chen et al. (2018) [14]	Cohort	NE	301	166	Medication nonadherence	2.854	1.357-6.005	Uncontrolled BP
Vilaubi et al. (2018)	Cohort	January 2004-May 2005	438	269	Guideline nonadherence	1.66	1.02-2.68	

Sarfo et al. (2016) [25]	Retrospective observational study	January 2012-June 2014	580	178	Stage of hypertension	1.31	1.17-1.47	Uncontrolled BP
					Use of antihypertension	1.3	1.06-1.60	

Nidhinandana et al. (2014) [26]	Retrospective observational study	February 2010-January 2011	558	349	Diabetes mellitus	0.18	0.11-0.29	Uncontrolled BP
					Stage 1 of HT	0.3	0.15-0.59	
					Stage 2 of HT	0.24	0.12-0.29	
					Use of antihypertension	0.53	0.33-0.85	

Dave et al. (2013) [16]	Case control	2004-2007	2663	1158	BP medications nonadherence	2.45	1.738-3.454	Uncontrolled SBP
					Age >55 years old	1.666	1.376-2.017	
					African-American race	1.558	1.272-1.908	
					Triglycerides (moderate risk)	1.49	1.164-1.907	
					Blood glucose	2.107	1.171-3.791	
				607	Age >55 years old	0.461	0.367-0.579	Uncontrolled DBP
					African-American race	2.173	1.708-2.765	
					Gender (male)	1.953	1.543-2.472	
					BP medications nonadherence	2.242	1.580-3.183	
					History of atrial fibrillation	0.477	0.242-0.938	
					Smoker	1.376	1.029-1.842	

Bhatt et al. (2016) [17]	Cohort	January 2003-October 2007	30018	5587	Atrial fibrillation	1.87	1.59-2.21	Uncontrolled SBP

Ofedal et al. (2018)	Cohort	September 2010-August 2015	385	104	Age	2.22	1.27-3.89	Uncontrolled BP
					BMI	1.14	1.08-1.20	
					Obesity	2.65	1.59-4.4	
					Pulse wave velocity	1.39	1.21-1.159	
					Carotid artery plaque	5	2.06-12.15	
					Metabolic syndrome	2.06	1.25-3.42	
					Diabetes	2.34	1.21-4.53	
					Number of antihypertensive drug	1.83	1.46-2.30	
					HDL cholesterol	0.51	0.31-0.84	
					Triglycerides	1.45	1.14-1.86	
					Fasting blood glucose	1.26	1.07-1.49	
					HbA1c	1.47	1.08-2.01	

NE: not explained; BP: blood pressure; SBP: systolic blood pressure; DBP: diastolic blood pressure.

## Data Availability

All data is attached in the table, and the data analysis has been shown in the figure.
